# Multi-modal data combination strategy based on chest HRCT images and PFT parameters for intelligent dyspnea identification in COPD

**DOI:** 10.3389/fmed.2022.980950

**Published:** 2022-12-21

**Authors:** Yingjian Yang, Ziran Chen, Wei Li, Nanrong Zeng, Yingwei Guo, Shicong Wang, Wenxin Duan, Yang Liu, Huai Chen, Xian Li, Rongchang Chen, Yan Kang

**Affiliations:** ^1^College of Medicine and Biological Information Engineering, Northeastern University, Shenyang, China; ^2^College of Health Science and Environmental Engineering, Shenzhen Technology University, Shenzhen, China; ^3^School of Applied Technology, Shenzhen University, Shenzhen, China; ^4^Department of Radiology, The First Affiliated Hospital of Guangzhou Medical University, Guangzhou, China; ^5^Shenzhen Institute of Respiratory Diseases, Shenzhen People's Hospital, Shenzhen, China; ^6^The Second Clinical Medical College, Jinan University, Guangzhou, China; ^7^The First Affiliated Hospital, Southern University of Science and Technology, Shenzhen, China; ^8^Engineering Research Centre of Medical Imaging and Intelligent Analysis, Ministry of Education, Shenyang, China

**Keywords:** dyspnea identification, COPD, multi-modal data, combination strategy, PFT parameters, lung radiomics features, 3D CNN features, machine learning

## Abstract

**Introduction:**

Because of persistent airflow limitation in chronic obstructive pulmonary disease (COPD), patients with COPD often have complications of dyspnea. However, as a leading symptom of COPD, dyspnea in COPD deserves special consideration regarding treatment in this fragile population for pre-clinical health management in COPD. Methods: Based on the above, this paper proposes a multi-modal data combination strategy by combining the local and global features for dyspnea identification in COPD based on the multi-layer perceptron (MLP) classifier.

**Methods:**

First, lung region images are automatically segmented from chest HRCT images for extracting the original 1,316 lung radiomics (OLR, 1,316) and 13,824 3D CNN features (O3C, 13,824). Second, the local features, including five selected pulmonary function test (PFT) parameters (SLF, 5), 28 selected lung radiomics (SLR, 28), and 22 selected 3D CNN features (S3C, 22), are respectively selected from the original 11 PFT parameters (OLF, 11), 1,316 OLR, and 13,824 O3C by the least absolute shrinkage and selection operator (Lasso) algorithm. Meantime, the global features, including two fused PFT parameters (FLF, 2), six fused lung radiomics (FLR, 6), and 34 fused 3D CNN features (F3C, 34), are respectively fused by 11 OLF, 1,316 OLR, and 13,824 O3C using the principal component analysis (PCA) algorithm. Finally, we combine all the local and global features (SLF + FLF + SLR + FLR + S3C + F3C, 5+ 2 + 28 + 6 + 22 + 34) for dyspnea identification in COPD based on the MLP classifier.

**Results:**

Our proposed method comprehensively improves classification performance. The MLP classifier with all the local and global features achieves the best classification performance at 87.7% of accuracy, 87.7% of precision, 87.7% of recall, 87.7% of F1-scorel, and 89.3% of AUC, respectively.

**Discussion:**

Compared with single-modal data, the proposed strategy effectively improves the classification performance for dyspnea identification in COPD, providing an objective and effective tool for COPD management.

## 1. Introduction

Chronic obstructive pulmonary disease (COPD) is a common lung disease characterized by persistent airflow limitation (1–3). Because of this characterization, patients with COPD often have complications of dyspnea (4). However, as a leading symptom of COPD (5), dyspnea in COPD deserves special consideration regarding treatment in this fragile population for pre-clinical health management in COPD. Furthermore, multi-modal biomedical data combination has been a hot research topic for facilitating precision and/or personalized medicine (6, 7). Therefore, multi-modal biomedical data combination is also crucial for pre-clinical health management in dyspnea caused by COPD.

Pulmonary function test (PFT) and computed tomography (CT) have become indispensable for COPD assessment and diagnosis. The PFT and CT have their own advantages in diagnosing and evaluating COPD and are complementary. Compared with CT, PFT is a non-invasive way to diagnose COPD from stage 0 to IV, according to Global Initiative for Chronic Obstructive Lung Disease (GOLD) criteria accepted by the American Thoracic Society and the European Respiratory Society (3). Specifically, the forced expiratory volume in 1 s/forced vital capacity (FEV_1_/FVC) and FEV_1_% predicted in PFT are the gold standards for diagnosing the COPD stage. Meanwhile, in patients with COPD, forced inspiration, particularly the assessment of FTV_1_, yields objective information that correlates closely with subjective dyspnea ratings after bronchodilator inhalation (8). In addition, compared with PFT, CT images can reflect the change in the lung tissue of COPD patients. Thus, CT has been regarded as the most effective modality for characterizing and quantifying COPD (9). Specifically, chest CT images can indicate that the patients have suffered from mild lobular central emphysema and reveal decreased exercise tolerance in smokers without airflow limitations in their PFT results (3, 10). In addition, chest CT images also can quantitatively analyze the bronchial, airway disease, emphysema, and vascular problems in COPD patients by measuring the parameters of the bronchi and vasculature (3). Based on the above, chest CT images should provide more imaging information for dyspnea identification in COPD.

Radiomics was proposed to mine more information from medical images using advanced feature analysis in 2007 for extracting more information from medical images (11). However, because the lesions as the region of interest (ROI) are diffusely distributed in the lungs, radiomics in COPD develops more slowly than other lung diseases, such as lung cancer or pulmonary nodules (12). With the significant progress of CT imaging technology, high-resolution CT (HRCT) imaging has become an effective method for the quantitative analysis of COPD (3, 12). However, quantitative analysis of bronchial and vascular blood flow is still limited by HRCT imaging resolution. Furthermore, it is challenging to automatically, semi-automatically, or manually segment small trachea (such as small airways) and blood vessels from chest HRCT images (13–15). In essence, COPD results from the characteristic pathological changes of the lung region, including the peripheral airway, parenchyma, and vessels. Therefore, lung imaging features extracted based on the lung region have been used for COPD analysis (3, 12). Therefore, it is reasonable for dyspnea identification based on lung region HRCT images and effectively avoids the limitations of challenging segmentation tasks of small airways and vessels, which is conducive to clinical application. Besides, the value of lung radiomics features extracted from lung region HRCT images in COPD assessment has also been confirmed (16).

There are potential applications of radiomics features in COPD, particularly for the diagnosis, treatment, and follow-up of COPD and future directions of radiomics features in COPD (17). Currently, lung radiomics features have been widely used for COPD stage classification (3, 12), COPD survival prediction (18, 19), COPD presence prediction (20), COPD exacerbations (21), COPD early decision (22), and analysis of COPD and resting heart rate (3). However, radiomics features are extracted from medical images by specific calculation equations, preset types of images, and preset classes, limiting the forms of radiomics features. Convolutional neural networks (CNN) based on images for classification also rapidly developed (23). Features extracted from medical images based on the CNN model will compensate for the limitations of radiomics features. Therefore, deep CNN features extracted from lung region HRCT images should be paid attention to improve the classification performance for facilitating precision and/or personalized medicine.

Dyspnea, one of COPD's main symptoms, is currently assessed with the Modified British medical research council (mMRC) questionnaire (24). The mMRC scale, the most common validated scale to assess dyspnea for COPD patients in daily living, is used to assess the dyspnea scale (25). However, the mMRC lacks objectivity in identifying dyspnea. The accuracy of the mMRC depends on the understanding and cooperation attitude of the evaluator. Based on the above, previous works identified dyspnea based on physiological signals. For example, mild dyspnea is detected from pairs of speech recordings, achieving an accuracy of about 74% (26). Besides, respiratory Symptoms are automatically detected using a low-power multi-input CNN processor, achieving an accuracy of 87.3% on dyspnea identification (27). However, dyspnea identification in COPD remains lacking research, especially clinically applying for pre-clinical health management in COPD using multi-modal data.

Above, we summarize the advantages and disadvantages of PFT and HRCT. However, integrating the advantages of the PFT parameters, the lung radiomics features, and CNN features is crucial for dyspnea identification. Therefore, this paper proposes a multi-modal data combination strategy for dyspnea identification in COPD based on the multi-layer perceptron (MLP) classifier, providing an objective and effective model of dyspnea identification. Our contributions in this paper are briefly described as follows:

1. We settle the problem that minor PFT parameters are easily overwhelmed by a large number of lung radiomics features and CNN features;
2. Further, inspired by CNN, we propose a combination strategy by combining the local and global features of the PFT parameters, the lung radiomics, and CNN features for improving the classification performance;
3. Last, our proposed combination strategy based on the MLP classifier achieves the best classification performance at 87.7% of accuracy, 87.7% of precision, 87.7% of recall, 87.7% of F1-score, and 89.3% of AUC, which may become an objective and effective tool for pre-clinical health management in COPD.

## 2. Materials and methods

This section details our study cohort and methodology, including the selection flow of 404 subjects, the dyspnea distribution of the subjects at different GOLD in our study cohort, and the framework of the proposed method.

### 2.1. Materials

This study had approved by the ethics committee in the national clinical research center of China's respiratory diseases. In addition, all subjects have been provided written informed consent by the first affiliated hospital of Guangzhou medical university before chest high-resolution computed tomography (HRCT) scans, PFT, and mMRC scale inquisition.

**Figure 1:**
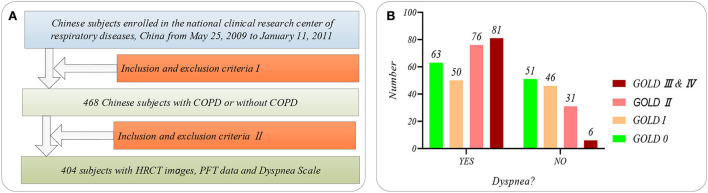
The subjects' selection flow diagram and dyspnea distribution in this study. **(A)** The subjects' selection flow diagram, including the enrollment and the inclusion and exclusion criteria I and II; **(B)** Dyspnea distribution of the subjects at GOLD 0-III&IV.

Figure 1 shows the selection flow of 404 subjects aged 40–79 in our study cohort and the dyspnea distribution of the subjects at different GOLD in our study cohort. Figure 1A shows the selection flow of 404 subjects in our study cohort. Specifically, Chinese participants are enrolled by the national clinical research center of respiratory diseases, China, from May 25, 2009, to January 11, 2011. Four hundred sixty-eight Chinese subjects participated in the study after being strictly selected by the inclusion and exclusion criteria I (28). More detailed inclusion and exclusion criteria I can also be found in our previous study (22). First, the 468 subjects were asked to undergo PFT and chest HRCT scans (TOSHIBA, kVp:120 kV, X-ray tube current:40 mA, slice thinkness:1.0 mm) at the full inspiration state. Then, 404 subjects are strictly selected from the 468 subjects by the inclusion and exclusion criteria II. The inclusion and exclusion criteria II requires that every subject meets the following two requirements simultaneously: (1) that subject with the chest HRCT images and PFT parameter; (2) the time of the chest HRCT images, PFT parameters, and mMRC scale on the same day. Normal ordinary people always have shortness of breath during strenuous exercise. Therefore, if shortness of breath occurs only during strenuous exercise (mMRC score of 0), it is considered that there is no dyspnea. Otherwise, it is considered that the subjects suffered from dyspnea (mMRC score of 1–4).

Besides, Figure 1B shows the dyspnea distribution of the subjects at different GOLD. Our study cohort includes 254 subjects who suffered from dyspnea and 150 subjects without dyspnea. Eleven PFT parameters (OLF, 11) include diffusing capacity for carbon monoxide (DL_CO_, mmol/ kPa x min), FEV_1_ (L), FEV_1_ after (FEV_1__AFT, L), FVC, FVC after (FEV_1__ AFT, L), functional residual capacity (FRC, L), inspiratory capacity IC (L), FEV_1_/FVC (%), Carbon Monoxide Corr for Alveolar (KCO_BP), residual volume (RV, L), and total lung capacity (TLC, L), referring to the ATS/ERS standard (American Thoracic Society 2005) (29). Statistical information on the PFT parameters is available in Supplementary Table S1 of Supplementary material.

### 2.2. Methods

**Figure 2:**
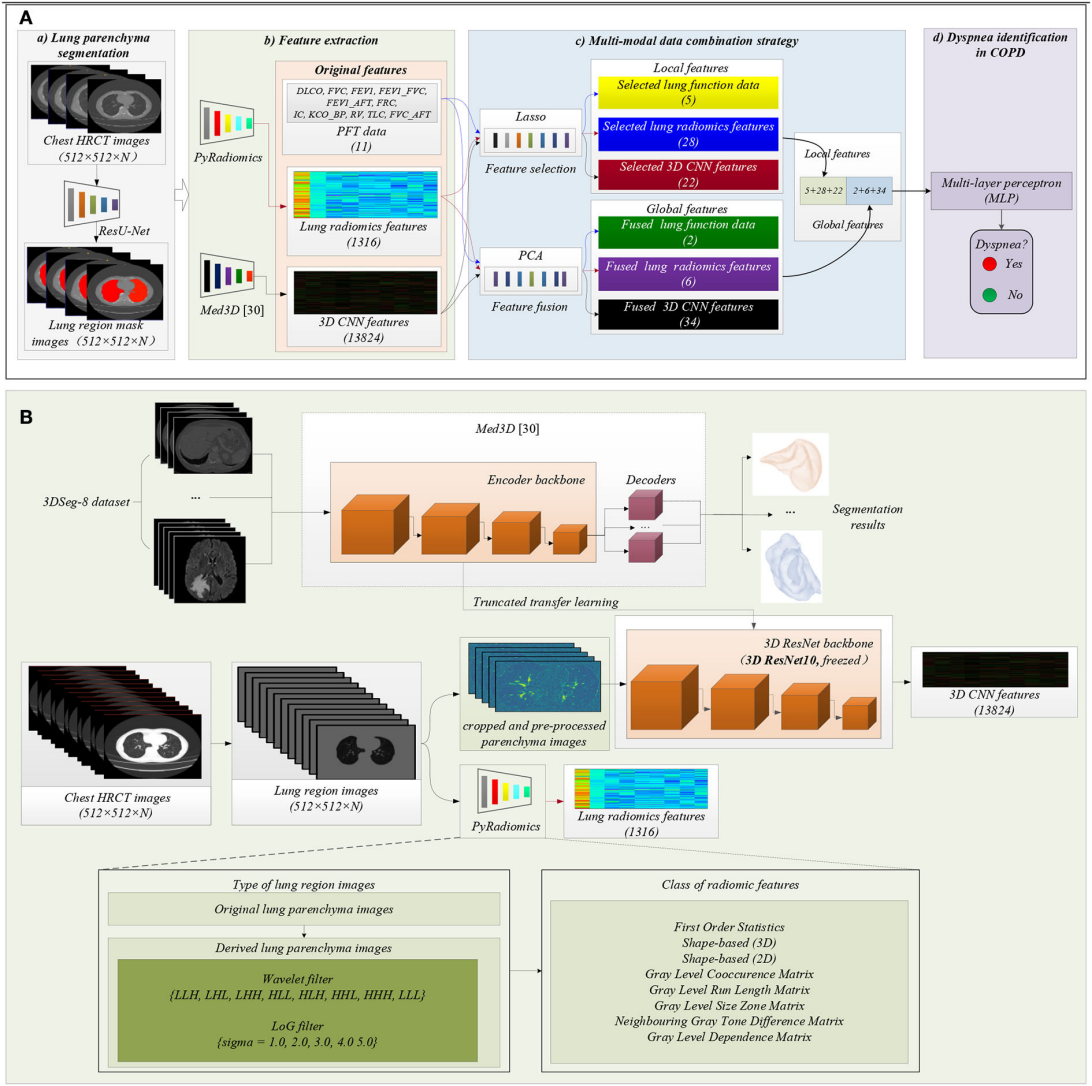
The framework of the proposed method. **(A)** The flow chart of the proposed method: (a) Lung region (ROI) segmentation; (b) Features extraction; (c) Multi-modal data combination strategy; (d) Dyspnea identification in COPD based on MLP classifier; **(B)** Detailed process of features extraction, including lung radiomics features and 3D CNN features.

Figure 2 shows the proposed method in this study. The main idea of the proposed method in this paper is to combine PFT parameters and chest HRCT images for intelligent dyspnea identification in COPD based on machine learning (ML) classifiers.

#### 2.2.1. Lung region segmentation

Figure 2A(a) shows that lung region mask images with red color are automatically segmented from the 404 sets of chest HRCT images using a state-of-the-art of ResU-Net (30). The ResU-Net trained by human chest CT images with different lung diseases is a robust and standard segmentation model of pathological lungs (12, 30). The architecture of the ResU-Net has been described in detail in our previous paper (31). In addition, 404 sets of lung region mask images have been checked and modified by experienced radiologists.

#### 2.2.2. Feature extraction

Figure 2A(b) shows that the two standard models PyRadiomics (32) and pre-trained Med3D (33) are selected to effectively and comprehensively extract the imaging features of lung region HRCT images. First, the 404 sets of the lung region HRCT images with the Hounsfield unit (HU) are obtained based on the lung region mask images and their chest HRCT images (34). Then, lung radiomics and 3D CNN features are separately extracted from the lung region HRCT images based on PyRadiomics and pre-trained Med3D. Finally, 1,316 original lung radiomics (OLR, 1,316) and 13,824 3D CNN features (O3C, 13,824) are obtained per subject.

Figure 2B details the feature extraction process of 1,316 OLR. Specifically, the lung region HRCT images with HU separately are filtered by wavelet filter and Laplacian of Gaussian filter (LoG) filter, generating the derived images. Then, the lung region HRCT images and their derived images are used to extract 1,316 OLR based on PyRadiomics. Please refer to our previous study (3, 12, 22) for a more detailed description of lung radiomics extraction. In addition, the PyRadiomics is available on the website (https://pyradiomics.readthedocs.io/en/latest/index.html), and the website also has given detailed explanations of radiomics (3).

Besides, Figure 2B also details the feature extraction process of 13,824 O3C. Med3D, a heterogeneous 3D network, is to extract general medical 3D features by building a 3DSeg-8 dataset with diverse modalities, target organs, and pathologies (12, 33). A truncated transfer learning strategy is adopted to extract the 3D CNN features based on the pre-trained Med3D. Thus, only the encoder backbone (3D ResNet10) of pre-trained Med3D needs to be transferred to generate 13,824 O3C. First, we use the same method in Med3D to crop and pre-process the lung region HRCT images (280 × 400 × N'). Second, the cropped and pre-processed lung images generate the CNN feature maps (512 × 35 × 50 × 75). Third, higher-order CNN feature maps (512 × 3 × 3 × 3) are obtained based on the CNN feature maps (512 × 35 × 50 × 75) by 3D average pooling. Finally, the higher-order CNN feature maps (512 × 3 × 3 × 3) per subject are flattened into 13,824 O3C (512 × 3 × 3 × 3 = 13,824).

Before generating the CNN feature maps (512 × 35 × 50 × 75), the cropped and pre-processed lung images need to normalize the lung region and generate random values outside the lung region in accord with Gaussian distribution. Specifically, the mathematical expression of normalization is given by Eq. (1).


(1) $$x^{\prime }=\frac{x-\bar{x}}{\sigma }$$

where *x*, $\bar{x}$, and σ are the HU value, mean, and mean square deviation of cropped and pre-processed lung images, respectively; *x*′ is the normalized value of cropped and pre-processed lung images.

#### 2.2.3. Multi-modal data combination strategy

Figure 2A(c) details the process of the proposed multi-modal data combination strategy in this paper. Inspired by CNN, a combination strategy by combining the local and global features of the PFT parameters, lung radiomics, and 3D CNN features is proposed for improving the classification performance.

The local and global features of 11 OLF, 1,316 OLR, and 13,824 O3C are available in Supplementary Tables S2–7 of Supplementary material.

First, the local features are respectively selected from 11 OLF, 1,316 OLR, and 13,824 O3C by the least absolute shrinkage and selection operator (Lasso) algorithm (35), which has been proved to improve classification performance (3). A standard python package LassoCV (definition in Python 3.6), with 10 fold cross-validation, is performed in this paper. Subsequently, the local features are selected, including five selected PFT parameters (SLF, 5), 28 selected lung radiomics features (SLR, 28), and 22 selected 3D CNN features (S3C, 22). Second, global features of 11 OLF, 1,316 OLR, and 13,824 O3C are respectively fused by the principal component analysis (PCA) algorithm (a classic algorithm for reducing the number of dimensions) with a general 95% contribution (36). A standard python package sklearn.decomposition.PCA(svd_solver='auto') (definition in Python 3.6) is performed in this paper. Subsequently, the global features are fused, including two fused PFT parameters (FLF, 2), six fused lung radiomics features (FLR, 6), and 34 fused 3D CNN features (F3C, 34). Finally, all the local and global features (SLF + FLF + SLR + FLR + S3C + F3C, 5 + 2 + 28 + 6 + 22 + 34) are combined as the variables for dyspnea identification in COPD.

The mathematical expression of the Lasso algorithm (3, 12, 22, 35) is given by Equation (2),


(2) $$arg\;min\left\{\sum_{i=1}^{n}{\left(y_{\text{i}}-\beta_{0}-\sum_{j=1}^{p}\beta_{j}x_{ij}\right)}^{2}+\lambda \sum_{j=0}^{p}\left|\beta_{j}\right|\right\}$$

where *x*_*ij*_ is the value of the independent variable (OLF: 404 × 11 subjects, OLR: 404 × 1,316 subjects, or O3C: 404 × 13,824 subjects) after a normalization operation (Min-max normalization) (3). *y*_*i*_ is the value of the dependent variable (subjects with dyspnea or without dyspnea), λ is the penalty parameter (λ ≥0), β_*j*_ is the regression coefficient, *i*∈[1, n], and *j*∈[0, *p*].

The detailed fused process of the PCA with the singular value decomposition (SVD) algorithm (36) is introduced in this paper. First, a feature matrix *A*_*m*×*n*__=_ (*a*_1_,*a*,*a*_3_,…,*a*_*n*_) is constructed by 404 subjects with their features (OLF: 404 × 11 subjects, OLR: 404 × 1,316 subjects, or O3C: 404 × 13,824 subjects). Second, the eigenvalues of the feature matrix *A*_*m*×*n*_ are obtained by the SVD algorithm (Eq. (3)-(4)). Third, Normalize the eigenvalues, rank the normalized eigenvalues in the order of large size, and determine the corresponding eigenvalues (λ_1_,λ_2_,λ_3_,…,λ_*k*_) with their 95% accumulation. Then, the eigenvectors are calculated based on the corresponding eigenvalues (λ__1_→_ξ_1_,λ__2_→_ξ_2_,λ__3_→_ξ_3_,…,λ__*k*_→_ξ_*k*_) used to construct the transformation matrix *P*_*k*×*n*__=_ (ξ_1_, ξ_2_, ξ_3_,…, ξ_*k*_) _*k*×*n*_. Last, the fused features *B*_*m*×*k*_ are obtained based on the feature matrix *A*_*m*×*n*_ and the transformation matrix *P*_*k*×*n*_ using Equation (4).


(3) $$\begin{array}{l} A^{T}A={\left(U\text{Σ}V^{T}\right)}^{T}U\text{Σ}V^{T}=V{\text{Σ}}^{T}U^{T}U\text{Σ}V^{T}\\ \;=V{\text{Σ}}^{T}\text{Σ}V^{T}=V{\text{Σ}}^{2}V^{T} \end{array}$$

(4) $$(\lambda_{1},\lambda_{2},\lambda_{3},\ldots ,\lambda_{k})=\left(\sqrt{\sigma_{1}},\sqrt{\sigma_{2}},\sqrt{\sigma_{3}},\ldots ,\sqrt{\sigma_{k}}\right)$$

(5) $$\begin{array}{l} B_{m}\times k=(b_{1},b_{2},b_{3},......b_{k})=A_{m\times n}P_{k\times n}^{T}\\ \;\;\;\;\;\;\;\;\;={\left(a_{1},a,a_{3},....a_{n}\right)}_{\left(m\times n\right)}\\ \;\;\;\;\;\;\;\;\;\;\;{\left(\xi_{1},\xi_{2},\xi_{3},.....,\xi_{k}\right)}_{k\times n}^{T} \end{array}$$

where *U*_m×m_ and *V*_n×n_ are the orthogonal matrices, ∑_*m*×*n*_ = (σ_1_, σ_2_, σ_3_,…, σ_*k*_) is the diagonal matrix, and σ_*i*_ is the i^th^ eigenvalues of the matrix *A*^*T*^*A*.

#### 2.2.4. Dyspnea identification in COPD

Early MLP classifier is a linear model, which can only handle simple binary classification and is difficult to analyze complex non-linear problems (37). However, its non-linear expression ability has been effectively improved by introducing hidden layers and activation functions. Currently, the MLP classifier is widely used in machine learning, pattern recognition, and other fields (38–41).

Figure 2A(c) shows that the MLP classifier based on all the local and global features is used to identify dyspnea in COPD. A standard python package sklearn.neural_network. MLP classifier (definition in Python 3.6) is performed to identify dyspnea. The parameters in the package MLP classifier are set: hidden_layer_sizes=(256,128,64), activation='tanh', solver='adam', alpha=0.0001, tol=0.0005, and max_iter=1000, respectively.

#### 2.2.5. Experiments

**Figure 3:**
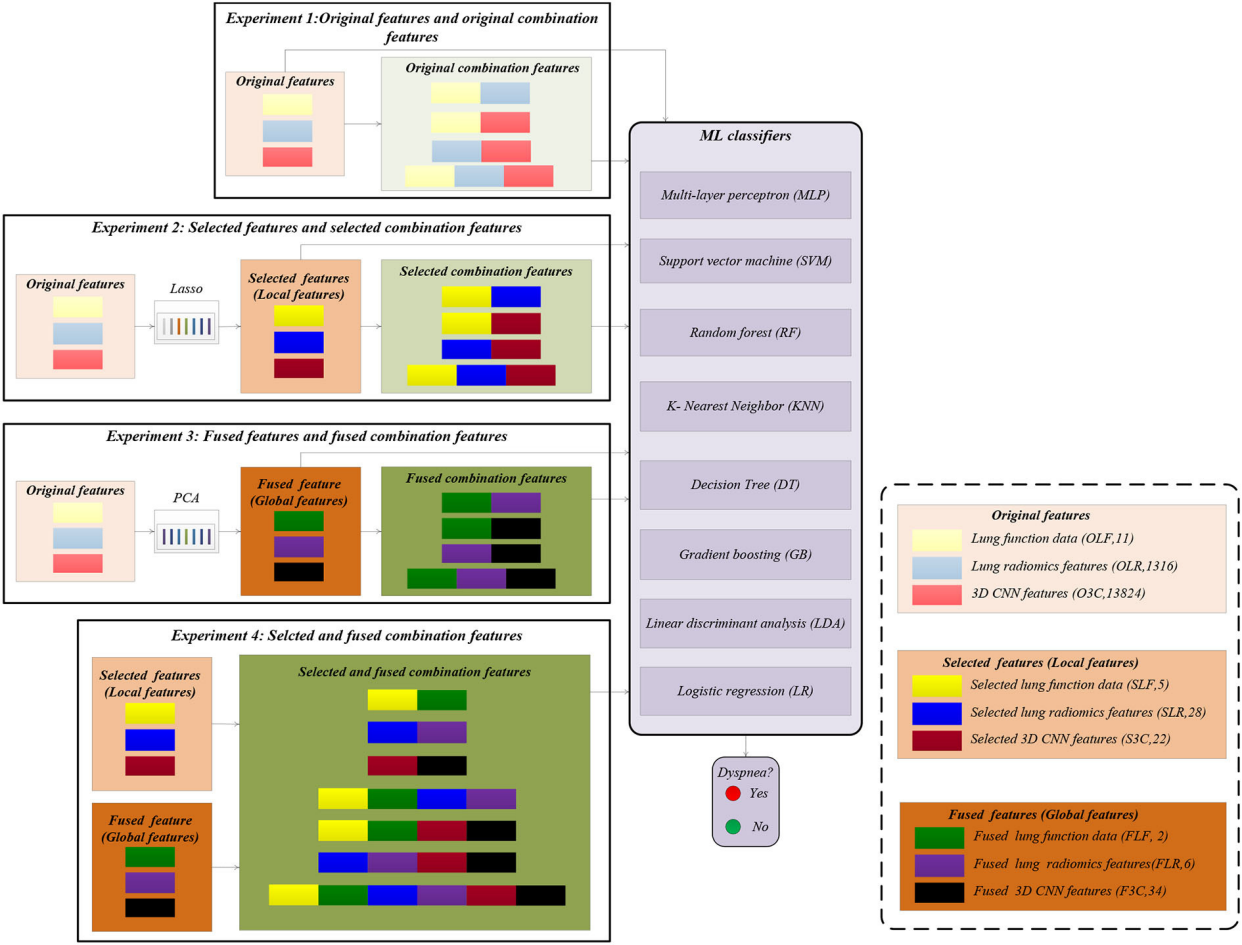
Experimental design in this paper.

Figure 3 shows the experimental design in this paper. Our experiment includes four experiments (Experiments 1–4) to verify the effectiveness of our proposed method. Previous studies used six classical machine learning (ML) classifiers to complete the COPD classification task (3). The six classical ML classifiers include MLP, support vector machine (SVM) (42), random forest (RF) (43), decision tree (DT) (44), gradient boosting (GB) (45), and linear discriminant analysis (LDA) (46). Based on the six above ML classifiers, K-nearest neighbor (KNN) (47) and logistic regression (LR) (48) are further considered to compare the performance of dyspnea recognition models further. Therefore, eight classical ML classifiers, including MLP, SVM, RF, KNN, DT, GB, LDA, and LR, are adopted to identify dyspnea in COPD based on different features. The definitions and parameters of the eight classifiers are available in Supplementary Table S8 of Supplementary material.

First, the 404 subjects in our study cohort are divided into the training set (*n* = 323) and the test set (*n* = 81). Specifically, 113 subjects suffered from dyspnea, and 210 subjects were without dyspnea in the training set. The 27 subjects suffered from dyspnea, and 54 subjects were without dyspnea in the test set. Then, the standard python packages of eight ML classifiers (definition in Python 3.6) are trained based on the training set, respectively. Last, the trained models are separately used to identify dyspnea based on the test set, giving the evaluation metrics of the classification performance. Specifically, the evaluation metrics of the classification performance include accuracy, precision, recall, F1-score, and area under the curve of AUC.

In Experiment 1, the classification performances are obtained based on the eight classical ML classifiers with OLF (11), OLR (1,316), O3C (13,824), and their arbitrary combination, respectively. In Experiment 2, the classification performances are obtained based on the above classifiers with SLF (5), SLR (28), S3C (22), and their arbitrary combination, respectively. Similarly, in Experiment 3, the classification performances are obtained based on the above ML classifiers with FLF (2), FLR (6), F3C (34), and their arbitrary combination, respectively. Finally, in Experiment 4, the classification performances are obtained based on the above classifiers with SLF + FLF (5 + 2), SLR + FLR (28 + 6), S3C + F3C (22 + 34), SLF + FLF + SLR + FLR (5 + 2 + 28 + 6), SLF + FLF + S3C + F3C (5 + 2 + 22 + 34), SLR + FLR + S3C + F3C (28 + 6 + 22 + 34), and SLF + FLF + SLR + FLR + S3C + F3C (selected ALL + fused ALL, 5 + 2 + 28 + 6 + 22 + 34), respectively.

## 3. Results

**Figure 4:**
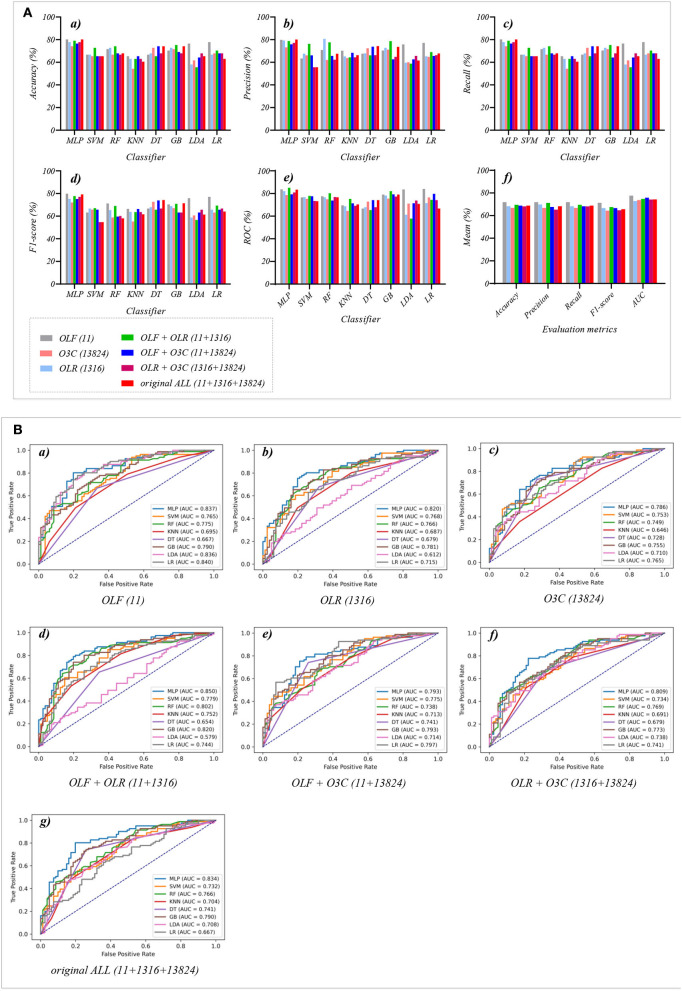
The evaluation metrics pictures and ROCs of eight classifiers with seven original classification features (three original features and four original combination features in Experiment 1). **(A)** The evaluation metrics pictures include **(a)** Accuracy, **(b)** Precision, **(c)** Recall, **(d)** F1-score, **(e)** AUC, and **(f)** Mean. **(B)** ROCs of the ML classifiers include **(a)** OLF (11), **(b)** OLR (1,316), **(c)** O3C (13,824), **(d)** OLF + OLR (11 + 1,316), **(e)** OLF + O3C (11 + 13,824), **(f)** OLR + O3C (1,316 + 13,824), **(g)** OLF + OLR + O3C (11 + 1,316 + 13,824).

**Figure 5:**
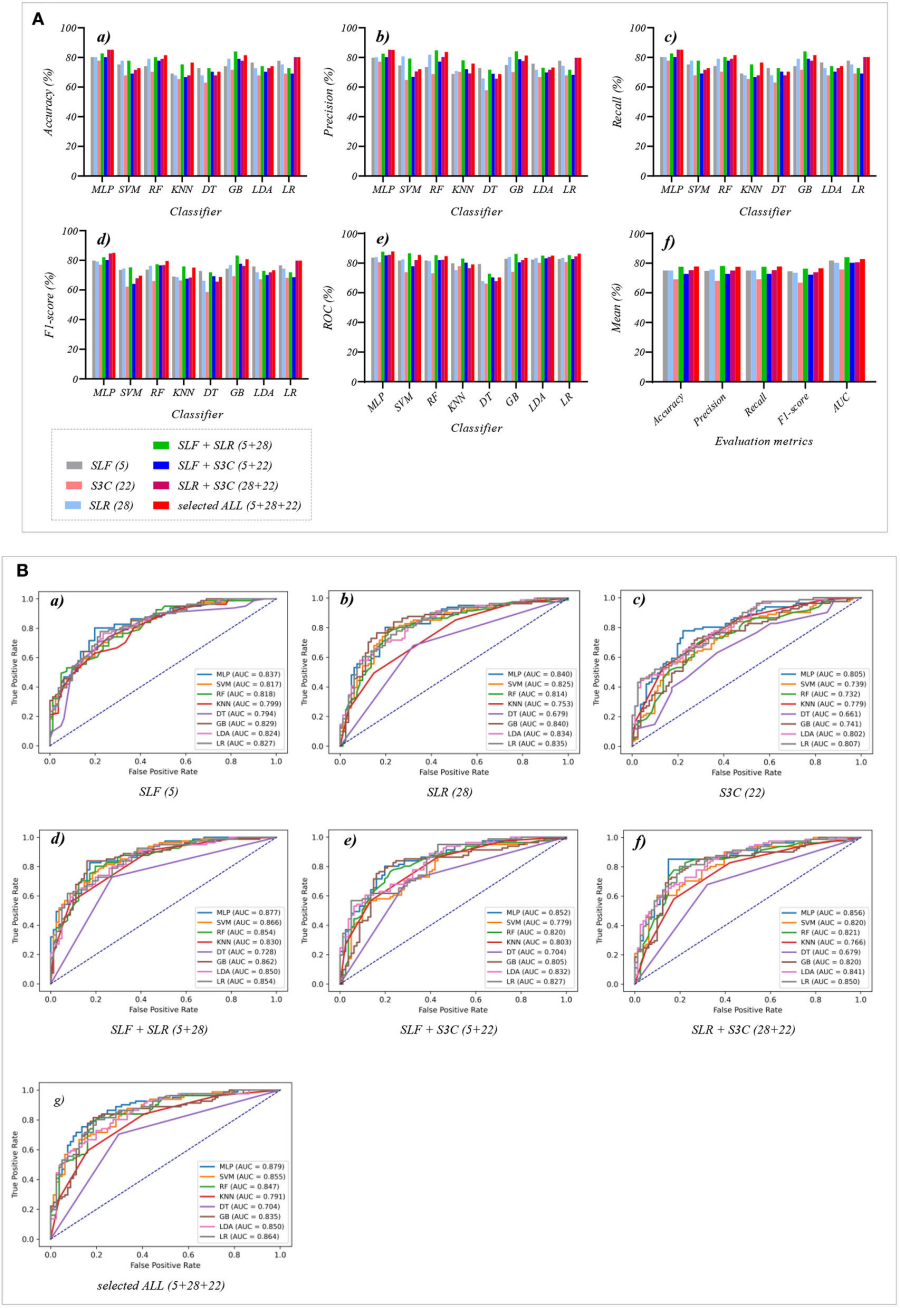
The evaluation metrics pictures and ROCs of eight classifiers with seven selected features (three selected features and four selected combination features in Experiment 2). **(A)** The evaluation metrics pictures include **(a)** Accuracy, **(b)** Precision, **(c)** Recall, **(d)** F1-score, **(e)** AUC, and **(f)** Mean. **(B)** ROCs of the ML classifiers include **(a)** SLF (5), **(b)** SLR (28), **(c)** S3C (22), **(d)** SLF + SLR (5 + 28), **(e)** SLF + S3C (5 + 22), **(f)** SLR + S3C (28 + 22), and **(g)** SLF + SLR + S3C (5 + 28 + 22).

**Figure 6:**
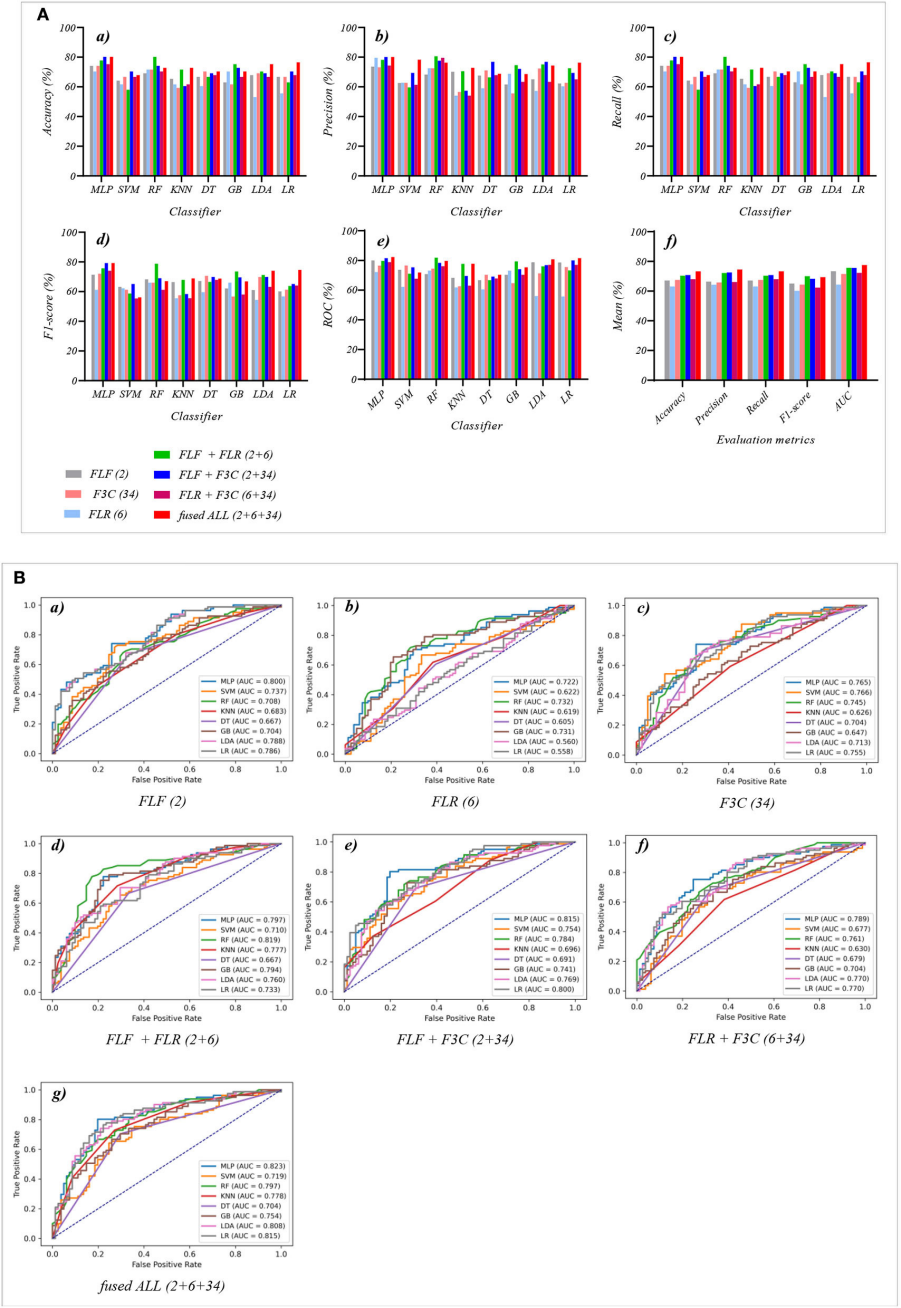
The evaluation metrics pictures and ROCs of eight classifiers with seven fused features (three fused features and four fused combination features in Experiment 3). **(A)** The evaluation metrics pictures include **(a)** Accuracy, **(b)** Precision, **(c)** Recall, **(d)** F1-score, **(e)** AUC, and **(f)** Mean. **(B)** ROCs of the ML classifiers include **(a)** FLF (2), **(b)** FLR (6), **(c)** F3C (34), **(d)** FLF + FLR (2 + 6), **(e)** FLF + F3C (2 + 34), **(f)** FLR + F3C (6 + 34), and **(g)** FLF + FLR + F3C (2 + 6 + 34).

**Figure 7:**
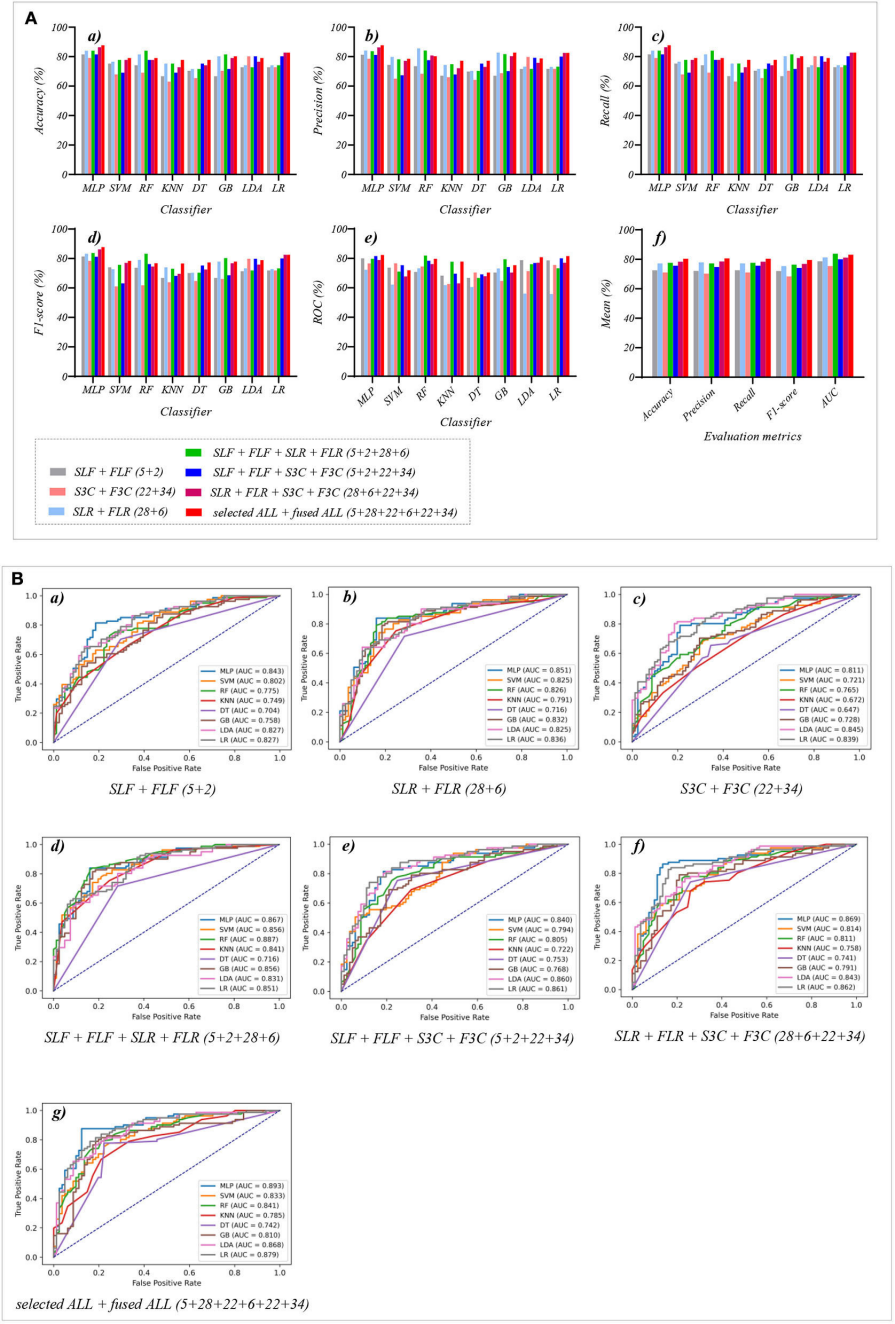
The evaluation metrics pictures and ROCs of eight classifiers with seven selected and fused combination features (Experiment 4). **(A)** The evaluation metrics pictures include **(a)** Accuracy, **(b)** Precision, **(c)** Recall, **(d)** F1-score, **(e)** AUC, and **(f)** Mean. **(B)** ROCs of the ML classifiers include **(a)** SLF + FLF (5+2), **(b)** SLR + FLR (28+6), **(c)** S3C + F3C (22+34), **(d)** SLF + FLF + SLR + FLR (5+2+28+6), **(e)** SLF + FLF + S3C + F3C (5+2+22+34), **(f)** SLR + FLR + S3C + F3C (28+6+22+34), and **(g)** SLF + FLF + SLR + FLR + S3C + F3C (selected ALL + fused ALL, 5+2+28+6+22+34).

This section reports the experimental results of the eight classical ML classifiers with different features. Specifically, Tables 1–**7** reports the experimental results of evaluation metrics. Figures 4–**7** visually shows these evaluation metrics, the mean value of evaluation metrics, and the receiver operating characteristic curve (ROC). In addition, the evaluation metric AUC in Tables 1–**7** is calculated from their ROCs.

**Table 1:** Evaluation metrics of the different classifiers with three original features (Experiment 1) on the test set.

**Classifier**	**Accuracy (%)**	**Precision (%)**	**Recall (%)**	**F1-score (%)**	**AUC (%)**
MLP	80.2^i^/77.8^ii^/74.1^iii^	79.8/79.2/73.2	80.2/77.8/74.1	79.8/75.2/72.0	83.7/82.0/78.6
SVM	76.5/66.7/65.4	63.4/67.6/66.1	66.7/66.7/65.4	63.2/66.7/65.7	76.5/76.8/75.3
RF	71.6/72.8/66.7	70.9/80.7/62.0	71.6/72.8/66.7	71.2/65.3/58.8	77.5/76.6/74.9
KNN	65.4/63.0/54.3	70.2/65.2/63.7	65.4/63.0/54.3	66.4/63.7/55.2	69.5/68.7/64.6
DT	66.7/67.9/72.8	67.6/67.9/72.4	66.7/67.9/72.8	66.7/67.9/72.6	66.7/67.9/72.8
GB	70.4/72.8/71.6	70.4/72.8/71.2	70.4/72.8/71.6	70.4/68.9/67.0	79.0/78.1/75.5
LDA	76.5/58.0/61.7	75.8/59.6/60.0	76.5/58.0/61.7	75.9/58.7/60.6	83.7/61.2/71.0
LR	77.8/66.7/67.9	77.1/65.3/64.8	77.8/66.7/67.9	77.0/65.7/63.2	84.0/71.5/76.5
(Mean)	71.9/68.2/66.8	71.9/69.8/66.7	71.9/68.2/66.8	71.3/66.6/64.4	77.6/72.9/73.7

i/ii/iii: OLF (11)/ OLR (1,316) / O3C (13,824).

### 3.1. The classification performance of original features and their combination features

Tables 1, 2 reports the experimental results of three original features and their combination features based on the eight classical ML classifiers in Experiment 1. Specifically, three original features include OLF (11), OLR (1,316), and O3C (13,824), and their combination features include OLF + OLR (11 + 1,316), OLF + O3C (11 + 13,824), OLR + O3C (1,316 + 13,824), and OLF + OLR + O3C (original ALL, 11 + 1,316 + 13,824).

**Table 2:** Evaluation metrics of the different classifiers with four original combination features (Experiment 1) on the test set.

** Classifier**	**Accuracy (%)**	**Precision (%)**	**Recall (%)**	**F1-score (%)**	**AUC (%)**
MLP	79.0^i^/76.5^ii^/77.8^iii^/80.2^iv^	78.8/75.9/77.1/80.1	79.0/76.5/77.8/80.2	77.7/75.1/77.0/79.2	85.0/79.3/80.9/83.4
SVM	72.8/65.4/65.4/65.4	76.3/66.1/55.6/55.6	72.8/65.4/65.4/65.4	67.0/65.7/54.7/54.7	77.9/77.5/73.4/73.2
RF	74.1/67.9/66.7/67.9	77.6/65.6/62.3/67.5	74.1/67.9/66.7/67.9	69.0/59.6/60.1/58.0	80.2/73.8/76.9/76.6
KNN	63.0/65.4/63.0/60.5	64.4/68.4/64.4/66.3	63.0/65.4/63.0/60.5	63.5/66.3/63.5/61.6	75.2/71.3/69.1/70.4
DT	65.4/74.1/67.9/74.1	66.1/73.8/66.3/74.3	65.4/74.1/67.9/74.1	65.7/73.9/66.7/74.2	65.4/74.1/67.9/74.1
GB	75.3/69.1/67.9/74.1	78.7/62.6/64.8/73.6	75.3/64.2/67.9/74.1	70.9/63.2/63.2/71.4	82.0/79.3/77.3/79.0
LDA	55.6/64.2/67.9/65.4	58.8/62.6/65.6/61.5	55.6/64.2/67.9/65.4	56.6/63.2/65.6/61.4	57.9/71.4/73.8/70.8
LR	70.4/67.9/67.9/63.0	69.0/65.6/66.3/67.8	70.4/67.9/67.9/63.0	69.3/65.6/66.7/64.0	74.4/79.7/74.1/66.7
(Mean)	69.5/68.8/68.1/68.8	71.2/67.6/65.3/68.3	69.4/68.2/68.1/68.8	67.5/66.6/64.7/65.6	74.8/75.8/74.2/74.3

i/ii/iii/iv: OLF + OLR (11 + 1,316)/ OLF + O3C (11 + 13,824)/ OLR + O3C (1,316 + 13,824)/ OLF + OLR + O3C (11 + 1,316 + 13,824).

Tables 1, 2 and Figures 4A(a–e) show that the MLP classifier performs better than other classifiers. Figure 4B shows the ROCs of the three single original features and their combination features based on the eight classical ML classifiers. Furthermore, the classification performance of the MLP classifier with OLF (11) is the best of the three original features, achieving 80.2% of accuracy, 79.8% of precision, 80.2% of recall, 79.8% of F1-scorel, and 83.7% of AUC. The classification performance of the MLP classifier with OLR is better than that of O3C, achieving 77.8% of accuracy, 79.2% of precision, 77.8% of recall, 75.2% of F1-scorel, and 82.0% of AUC. However, all original combination features have not improved the classification performance compared with single OLF (11). Specifically, the classification performance of the MLP classifier with OLF + OLR (11 + 1,316) performs best at AUC, achieving 85.0%. Other evaluation metrics of OLF + OLR (11 + 1,316) based on the MLP classifier are 79.0% of accuracy, 78.8% of precision, 79.0% of recall, and 77.7% of F1-scorel. Except for OLF + OLR (11 + 1,316), the MLP classifier with the original ALL (11 + 1,316 + 13,824) performs better than other original combination features at AUC, achieving 83.4%. Other evaluation metrics of original ALL (11 + 1,316 + 13,824) based on the MLP classifier are 80.2% of accuracy, 80.1% of precision, 80.2% of recall, and 79.2% of F1-scorel. Figure 4A(f) shows the mean evaluation metrics of all classifiers in Experiment 1, and the mean evaluation metrics of single original features OLF (11) are best.

### 3.2. The classification performance of selected features and their combination features

Tables 3, 4 reports the experimental results of three selected features and their combination features based on the eight classical ML classifiers in Experiment 2. Specifically, three selected features include SLF (5), SLR (28), and S3C (22), and their combination features include SLF + SLR (5 + 28), SLF + S3C (5 + 22), SLR + S3C (28 + 22), and SLF + SLR + S3C (selected ALL, 5 + 28 + 22).

**Table 3:** Evaluation metrics of the different classifiers with three selected features (Experiment 2) on the test set.

** Classifier**	**Accuracy (%)**	**Precision (%)**	**Recall (%)**	**F1-score (%)**	**AUC (%)**
MLP	80.2^i^/80.2^ii^/77.8^iii^	79.8/80.1/77.1	80.2/80.2/77.8	79.8/79.2/77.0	83.7/84.0/80.5
SVM	75.3/77.8/67.9	74.6/80.8/64.8	75.3/77.8/67.9	73.5/74.5/62.2	81.7/82.5/73.9
RF	74.1/79.0/70.4	73.5/81.8/68.8	74.1/79.0/70.4	73.7/76.3/66.0	81.8/81.4/73.2
KNN	69.1/67.9/65.4	68.9/70.8/70.2	69.1/67.9/65.4	69.0/68.7/66.4	79.9/75.3/77.9
DT	72.8/67.9/63.0	72.8/65.9/57.9	72.8/67.9/63.0	72.8/66.2/58.6	79.4/67.9/66.1
GB	74.1/79.0/71.6	74.9/80.3/70.1	74.1/79.0/71.6	74.4/76.8/69.3	82.9/84.0/74.1
LDA	76.5/72.8/67.9	75.8/71.7/66.8	76.5/72.8/67.9	75.9/71.9/67.2	82.4/83.4/80.2
LR	77.8/75.3/69.1	77.8/74.4/67.9	77.8/75.3/69.1	76.6/74.4/68.2	82.7/83.5/80.7
(Mean)	75.0/75.0/69.1	74.8/75.7/68.0	75.0/75.0/69.1	74.5/73.5/66.9	81.8/80.3/75.8

i/ii/iii: SLF (5)/ SLR (28)/ S3C (22).

**Table 4:** Evaluation metrics of the different classifiers with four selected combination features (Experiment 2) on the test set.

** Classifier**	**Accuracy (%)**	**Precision (%)**	**Recall (%)**	**F1-score (%)**	**AUC (%)**
MLP	82.7^i^/80.2^ii^/85.2^iii^/85.2^iv^	82.5/80.2/85.2/85.0	82.7/80.2/85.2/85.2	82.1/80.2/84.6/85.0	87.7/85.2/85.6/87.9
SVM	77.8/69.1/71.6/72.8	79.2/66.9/70.5/72.1	77.8/69.1/71.6/72.8	75.2/64.1/67.9/69.6	86.6/77.9/82.0/85.5
RF	80.2/77.8/79.0/81.5	84.8/77.2/80.3/83.7	80.2/77.8/79.0/81.5	77.4/76.6/76.8/79.5	85.4/82.0/82.2/84.7
KNN	75.3/66.7/67.9/76.5	78.0/72.0/69.2/75.9	75.3/66.7/67.9/76.5	75.9/67.6/68.4/75.1	83.0/80.3/76.6/79.1
DT	72.8/70.4/67.9/70.4	71.7/69.0/65.6/68.8	72.8/70.4/67.9/70.4	71.9/69.3/65.6/68.8	72.8/70.4/67.9/70.4
GB	84.0/79.0/77.8/81.5	84.1/78.8/77.6/81.3	84.0/79.0/77.8/81.5	83.2/77.7/76.2/80.7	86.2/80.5/82.0/83.5
LDA	74.1/70.4/72.8/74.1	73.0/69.9/71.7/73.2	74.1/70.4/72.8/74.1	72.9/70.1/71.9/73.3	85.0/83.2/84.1/85.0
LR	72.8/69.1/80.2/80.2	71.7/68.3/79.8/79.8	72.8/69.1/80.2/80.2	71.9/68.6/79.8/79.8	85.4/82.7/84.5/86.4
(Mean)	77.5/72.8/75.3/77.8	78.1/72.8/75.0/77.5	77.5/72.8/75.3/77.8	76.3/72.2/73.9/76.5	84.0/80.2/80.6/82.8

i/ii/iii/iv: SLF + SLR (5 + 28)/ SLF + S3C (5 + 22)/ SLR + S3C (28 + 22)/ SLF + SLR + S3C (5 + 28 + 22).

Table 3 and Figures 5A(a–e) show that the MLP classifier with three single-selected features performs better than other classifiers in Experiment 2. Figure 5B shows the ROCs of the three single-selected features and their combination features based on the eight classical ML classifiers. Furthermore, the MLP classifier with SLR (5) performs best, achieving 80.2% of accuracy, 80.1% of precision, 80.2% of recall, 79.2% of F1-score, and 84.0% of AUC. Compared with the classification performance of OLF (11) based on the MLP classifier, that of SLF (5) remains unchanged. However, the MLP classifier with SLR (28) and S3C (22) separately performs better than that with OLR (1,316) and O3C (13,824). Specifically, the classification performance of SLR (28) has improved by 2.4% of accuracy, 0.9% of precision, 2.4% of recall, 4.0% of F1-scorel, and 2.0% of AUC. On the other hand, the classification performance with S3C (22) has improved by 3.7% of accuracy, 3.9% of precision, 3.7% of recall, 5.0% of F1-scorel, and 1.9% of AUC.

Table 4 and Figures 5A(a–e) show that the MLP classifier with selected combination features SLF + SLR + S3C (5 + 28 + 22) performs best. Specifically, the SLF + SLR + S3C (selected ALL, 5 + 28 + 22) based on the MLP classifier performs best, achieving 85.2% of accuracy, 85.0% of precision, 85.2% of recall, 85.0% of F1-scorel, and 87.9% of AUC. Compared with the classification performance of the single-selected features based on the MLP classifier, the selected combination features based on the MLP classifier performs better. Specifically, compared with the best classification performance of the single-selected features SLR (28) based on the MLP classifier, that of the selected combination features SLF + SLR + S3C (selected ALL, 5+28+22) has improved by 5.0% of accuracy, 4.9% of precision, 5.0% of recall, 5.8% of F1-scorel, and 3.9% of AUC. Compared with the classification performance of the original combination features based on the MLP classifier shown in Table 2, that of the selected combination features based on the MLP classifier has been improved, as shown in Table 4. Specifically, compared with the classification performance of OLF + OLR (11+1,316) based on the MLP classifier, that of SLF + SLR (5+28) based on the MLP classifier has improved by 3.7% of accuracy, 3.7% of precision, 3.7% of recall, 4.4% of F1-scorel, and 2.7% of AUC. Compared with the classification performance of OLF + O3C (11 + 13,824) based on the MLP classifier, that of SLF + S3C (5 + 22) based on the MLP classifier has improved by 3.7% of accuracy, 4.3% of precision, 3.7% of recall, 5.1% of F1-scorel, and 5.9% of AUC. Compared with the classification performance of OLR + O3C (1,316 + 13,824) based on the MLP classifier, that of SLR + S3C (28 + 22) based on the MLP classifier has improved by 7.4% of accuracy, 8.1% of precision, 7.4% of recall, 7.6% of F1-scorel, and 4.7% of AUC. Compared with the classification performance of OLR + O3C (1,316 + 13,824) based on the MLP classifier, that of SLR + S3C (28 + 22) based on the MLP classifier has improved by 5.0% of accuracy, 4.9% of precision, 5.0% of recall, 5.8% of F1-scorel, and 4.5% of AUC.

Figure 5A(f) shows the mean evaluation metrics of all classifiers in Experiment 2. The mean evaluation metrics of ML classifiers based on selected combination features SLF + SLR (5 + 28) and selected ALL (5 + 28 + 22) perform better than the single-selected features. In addition, compared with the mean evaluation metrics of ML classifiers based on the original features and their combination features, that of ML classifiers based on the selected features and their combination features has been improved. Specifically, compared with the best mean evaluation metrics of OLF (11) (71.9% of mean accuracy, 71.9% of mean precision, 71.9% of mean recall, 71.3% of mean F1-score, and 77.6% of mean AUC) in Figure 4A(f), that of SLF + SLR (5 + 28) has improved by 5.6% of accuracy, 6.2% of precision, 5.6% of recall, 5.0% of F1-score, and 6.4% of AUC. Compared with the best mean evaluation metrics of OLF (11), that of selected ALL (5+28+22) has improved by 5.9% of accuracy, 5.6% of precision, 5.9% of recall, 5.0% of F1-score), and 5.2% of AUC.

### 3.3. The classification performance of fused features and their combination features based on different classifiers

Tables 5, 6 reports the experimental results of three fused features and their combination features based on the eight classical ML classifiers in Experiment 3. Specifically, three fused features include FLF (2), FLR (6), and F3C (34), and their combination features include FLF + FLR (2 + 6), FLF + F3C (2 + 34), FLR + F3C (6 + 34), and FLF + FLR + F3C (fused ALL, 2 + 6 + 34).

**Table 5:** Evaluation metrics of the different classifiers with three fused features (Experiment 3) on the test set, respectively.

** Classifier**	**Accuracy (%)**	**Precision (%)**	**Recall (%)**	**F1-score (%)**	**AUC (%)**
MLP	74.1^i^/70.4^ii^/74.1^iii^	73.6/79.5/73.2	74.1/70.4/74.1	71.4/61.2/72.0	80.0/72.2/76.5
SVM	64.2/61.7/66.7	62.6/62.8/62.7	64.2/61.7/66.7	63.2/62.2/61.3	73.7/62.2/76.6
RF	69.1/71.6/71.6	68.3/72.5/72.5	69.1/71.6/71.6	68.3/66.0/66.0	70.8/73.2/74.5
KNN	65.4/61.7/59.3	70.2/54.1/56.5	65.4/61.7/59.3	66.4/55.5/57.5	68.3/61.9/62.6
DT	66.7/60.5/70.4	67.6/59.0/71.0	66.7/60.5/70.4	67.1/59.6/70.6	66.7/60.5/70.4
GB	63.0/70.4/61.7	61.6/68.8/55.6	63.0/70.4/61.7	62.1/66.0/56.7	70.4/73.1/64.7
LDA	67.9/53.1/69.1	65.0/57.2/72.4	67.9/53.1/69.1	61.0/54.4/69.9	78.8/56.0/71.3
LR	66.7/55.6/66.7	62.3/60.4/62.7	66.7/55.6/66.7	60.1/56.8/61.3	78.6/55.8/75.5
(Mean)	67.1/63.1/67.5	66.4/64.3/65.8	67.1/63.1/67.5	65.0/60.2/64.4	73.4/64.4/71.5

i/ii/iii: FLF (2)/ FLR (6)/F3C (34).

**Table 6:** Evaluation metrics of the different classifiers with four fused combination features (Experiment 3) on the test set, respectively.

** Classifier**	**Accuracy (%)**	**Precision (%)**	**Recall (%)**	**F1-score (%)**	**AUC (%)**
MLP	77.8^i^/80.2^ii^/75.3^iii^/80.2^iv^	78.2/80.1/74.4/80.1	77.8/80.2/75.3/80.2	75.7/79.2/74.0/79.2	79.7/81.5/78.9/82.3
SVM	58.0/70.4/66.7/67.9	59.6/69.4/61.4/78.3	58.0/70.4/66.7/67.9	58.7/65.1/55.4/56.1	71.0/75.4/67.7/71.9
RF	80.2/74.1/70.4/72.8	80.6/77.6/79.5/76.3	80.2/74.1/70.4/72.8	78.8/69.0/61.2/67.0	81.9/78.4/76.1/79.7
KNN	71.6/60.5/61.7/72.8	70.5/57.4/54.1/72.8	71.6/60.5/61.7/72.8	67.9/58.4/55.5/68.9	77.7/69.6/63.0/77.8
DT	66.7/69.1/67.9/70.4	66.4/76.9/67.9/68.8	66.7/69.1/67.9/70.4	66.5/69.9/67.9/68.8	66.7/69.1/67.9/70.4
GB	75.3/72.8/66.7/70.4	74.6/72.1/63.4/68.6	75.3/72.8/66.7/70.4	73.5/69.9/58.0/66.9	79.4/74.1/70.4/75.4
LDA	70.4/69.1/66.7/75.3	75.1/76.9/63.4/74.4	70.4/69.1/66.7/75.3	71.2/69.9/63.2/74.0	76.0/76.9/77.0/80.8
LR	63.0/70.4/67.9/76.5	72.5/69.4/65.0/76.3	63.0/70.4/67.9/76.5	63.8/65.1/64.1/74.6	73.3/80.0/77.0/81.5
(Mean)	70.4/70.8/67.9/73.3	72.2/72.5/66.1/74.5	70.4/70.8/67.9/73.3	69.5/68.3/62.4/69.4	75.7/75.6/72.3/77.5

i/ii/iii/iv: FLF + FLR (2 + 6)/ FLF + F3C (2 + 34)/ FLR + F3C (6 + 34)/ FLF + FLR + F3C (2 + 6 + 34).

Table 5 and Figures 6A(a–e) show that the MLP classifier with three single-fused features performs better than other classifiers in Experiment 3. Figure 6B shows the ROCs of the three single-fused features and their combination features based on the eight classical ML classifiers. Furthermore, the MLP classifier with FLR (6) performs best at AUC, achieving 80.0%. Other evaluation metrics of FLR on the MLP classifier are 74.1% of accuracy, 73.6% of precision, 74.1% of recall, and 74.1% of F1-scorel. However, compared with the classification performance of single original features and single-selected features based on the MLP classifier in Tables 1, 3, the MLP classifier with single-fused features fails to improve the classification performance.

Table 6 and Figures 6A(a–e) show that FLF + FLR + F3C (fused ALL, 2 + 6 + 34) based on the MLP classifier also performs best at AUC, achieving 82.3%. Other evaluation metrics of fused ALL based on the MLP classifier are 80.2% of accuracy, 80.1% of precision, 80.2% of recall, and 79.2% of F1-scorel. Compared with the best classification performance of the single-fused features FLR based on the MLP classifier, that of fused ALL based on the MLP classifier has improved by 6.1% of accuracy, 6.5% of precision, 6.1% of recall, 5.1% of F1-scorel, and 2.3% of AUC. However, compared with the classification performance of original combination features and selected combination features based on the MLP classifier in Tables 2, 4, the fused combination features based on the MLP classifier fail to improve the classification performance.

Figure 6A(f) shows the mean evaluation metrics of all classifiers in Experiment 3. The mean evaluation metrics of fused combination features FLF + FLR + F3C (2 + 6 + 34) perform better than the single-fused features. However, compared with the best mean evaluation metrics of OLF (11) in Figure 4A(f) and SLF + SLR (5 + 28) / selected ALL (5 + 28 + 22) in Figure 5A(f), the fused combination features FLF + FLR + F3C (2 + 6 + 34) fail to improve the mean evaluation metrics.

### 3.4. The classification performance of the selected and fused combination features

Table 7 reports the experimental results of three selected and fused combination features based on the eight classical ML classifiers in Experiment 4. Specifically, seven selected and fused combination features include SLF + FLF (5 + 2), SLR + FLR (28 + 6), S3C + F3C (22 + 34), SLF + FLF + SLR + FLR (5 + 2 + 28 + 6), SLF + FLF + S3C + F3C (5 + 2 + 22 + 34), SLR + FLR + S3C + F3C (28 + 6 + 22 + 34), SLF + FLF + SLR + FLR + S3C + F3C (selected ALL + fused ALL, 5 + 2 + 28 + 6 + 22 + 34).

**Table 7:** Evaluation metrics of the different classifiers with seven selected and fused combination features (Experiment 4) on the test set.

** Classifier**	**Accuracy (%)**	**Precision (%)**	**Recall (%)**	**F1-score (%)**	**AUC (%)**
MLP	81.5^i^/84.0^ii^/79.0^iii^/84.0^iv^/81.5^v^/86.4^vi^/87.7^vii^	81.3/84.1/78.5/83.7/81.1/86.3/87.7	81.5/84.0/79.0/84.0/81.5/86.4/87.7	81.4/83.2/78.4/83.7/81.2/86.2/87.7	84.3/85.1/81.1/86.7/84.0/86.9/89.3
SVM	75.3/76.5/67.9/77.8/69.1/77.8/79.0	74.4/79.8/65.0/78.2/67.4/77.1/78.5	75.3/76.5/67.9/77.8/69.1/77.8/79.0	74.0/72.7/61.0/75.7/63.1/77.0/78.4	80.2/82.5/72.1/85.6/79.4/81.4/83.3
RF	74.1/81.5/69.1/84.0/77.8/77.8/79.0	73.5/85.5/68.4/84.1/77.6/80.8/80.3	74.1/81.5/69.1/84.0/77.8/77.8/79.0	73.7/79.0/61.8/83.2/76.2/74.5/76.8	77.5/82.6/76.5/88.7/80.5/81.1/84.1
KNN	66.7/75.3/63.0/75.3/69.1/72.8/77.8	67.0/74.4/66.0/74.9/67.9/72.1/77.2	66.7/75.3/63.0/75.3/69.1/72.8/77.8	66.8/74.0/63.9/73.0/68.2/69.6/76.6	74.9/79.1/67.2/84.1/72.2/75.8/78.5
DT	70.4/71.6/65.4/71.6/75.3/74.1/77.8	69.9/70.3/64.2/70.3/75.3/73.0/77.2	70.4/71.6/65.4/71.6/75.3/74.1/77.8	70.1/70.4/64.7/70.4/75.3/72.5/77.3	70.4/71.6/64.7/71.6/75.3/74.1/74.2
GB	66.7/80.2/70.4/81.5/71.6/79.0/80.2	67.0/82.7/68.8/81.7/70.2/80.3/82.7	66.7/80.2/70.4/81.5/71.6/79.0/80.2	66.8/77.9/66.0/80.3/68.6/76.8/77.9	75.8/83.2/72.8/85.6/76.8/79.1/81.0
LDA	72.8/74.1/80.2/72.8/80.2/76.5/79.0	71.6/73.2/79.8/71.7/79.2/75.8/78.8	72.8/74.1/80.2/72.8/80.2/76.5/79.0	71.4/73.3/79.8/71.9/79.8/75.9/78.9	82.7/82.5/84.5/83.1/86.0/84.3/86.8
LR	72.8/74.1/72.8/74.1/80.2/82.7/82.7	71.7/73.0/71.7/73.2/80.0/82.5/82.5	72.8/74.1/72.8/74.1/80.2/82.7/82.7	71.9/72.9/71.9/73.3/80.0/82.5/82.5	82.7/83.6/83.9/85.1/86.1/86.2/87.9
(Mean)	72.5/77.2/71.0/77.6/75.6/78.4/80.4	72.1/77.9/70.3/77.2/74.8/78.5/80.6	72.5/77.2/71.0/77.6/75.6/78.4/80.4	72.0/75.4/68.4/76.4/74.1/76.9/79.5	78.6/81.3/75.4/83.8/80.0/81.1/83.1

i/ii/iii/iv/v/vi/vii: SLF + FLF (5 + 2)/ SLR + FLR (28 + 6)/ S3C + F3C (22 + 34)/ SLF + FLF + SLR + FLR (5 + 2 + 28 + 6)/ SLF + FLF + S3C + F3C (5 + 2 + 22 + 34)/ SLR + FLR + S3C + F3C (28 + 6 + 22 + 34)/ SLF + FLF + SLR + FLR + S3C + F3C (selected ALL + fused ALL → our proposed strategy, 5 + 2 + 28 + 6 + 22 + 34).

Table 7 and Figures 7A(a–e) show that the MLP classifier with selected ALL+fused ALL (our proposed strategy, 5 + 2 + 28 + 6 + 22 + 34) performs best in Experiments 1–4. Figure 7B shows the ROCs of the seven selected and fused combination features based on the eight classical ML classifiers. Specifically, the MLP classifier with selected ALL + fused ALL (5 + 2 + 28 + 6 + 22 + 34) achieves 87.7% of accuracy, 87.7% of precision, 87.7% of recall, 87.7% of F1-scorel, and 89.3% of AUC. Compared with the best classification performance of the single original feature OLF (11) based on the MLP classifier in Experiment 1, the classification performance of the MLP classifier with our proposed strategy has improved by 7.5% of accuracy, 7.9% of precision, 7.5% of recall, 7.9% of F1-scorel, and 5.6% of AUC. Compared with the best classification performance of the selected combination feature selected ALL (5 + 28 + 22) based on the MLP classifier in Experiment 2, the classification performance of the MLP classifier with our proposed strategy has improved by 2.5% of accuracy, 2.9% of precision, 2.5% of recall, 2.7% of F1-scorel, and 1.4% of AUC. Compared with the best classification performance of the fused combination feature fused ALL (2+6+34) based on the MLP classifier in Experiment 3, the classification performance of the MLP classifier with our proposed strategy has improved by 7.5% of accuracy, 7.6% of precision, 7.5% of recall, 8.5% of F1-scorel, and 7.0% of AUC.

Figure 7A(f) shows the mean evaluation metrics of all classifiers in Experiment 4. The mean evaluation metrics of selected combination features SLF + FLF + SLR + FLR (5 + 2 + 28 + 6) perform best at mean AUC, achieving 83.8%. The mean AUC of selected ALL+fused ALL is marginally lower than that of SLF + FLF + SLR + FLR, achieving 83.1%. However, other mean evaluation metrics of selected ALL+fused ALL perform best, achieving 80.4% of accuracy, 80.6% of precision, 80.4% of recall, and 79.5% of F1-scorel. The mean evaluation metrics of selected ALL+fused ALL are far superior to the best mean evaluation metrics in Experiments 1–4.

**Figure 8:**
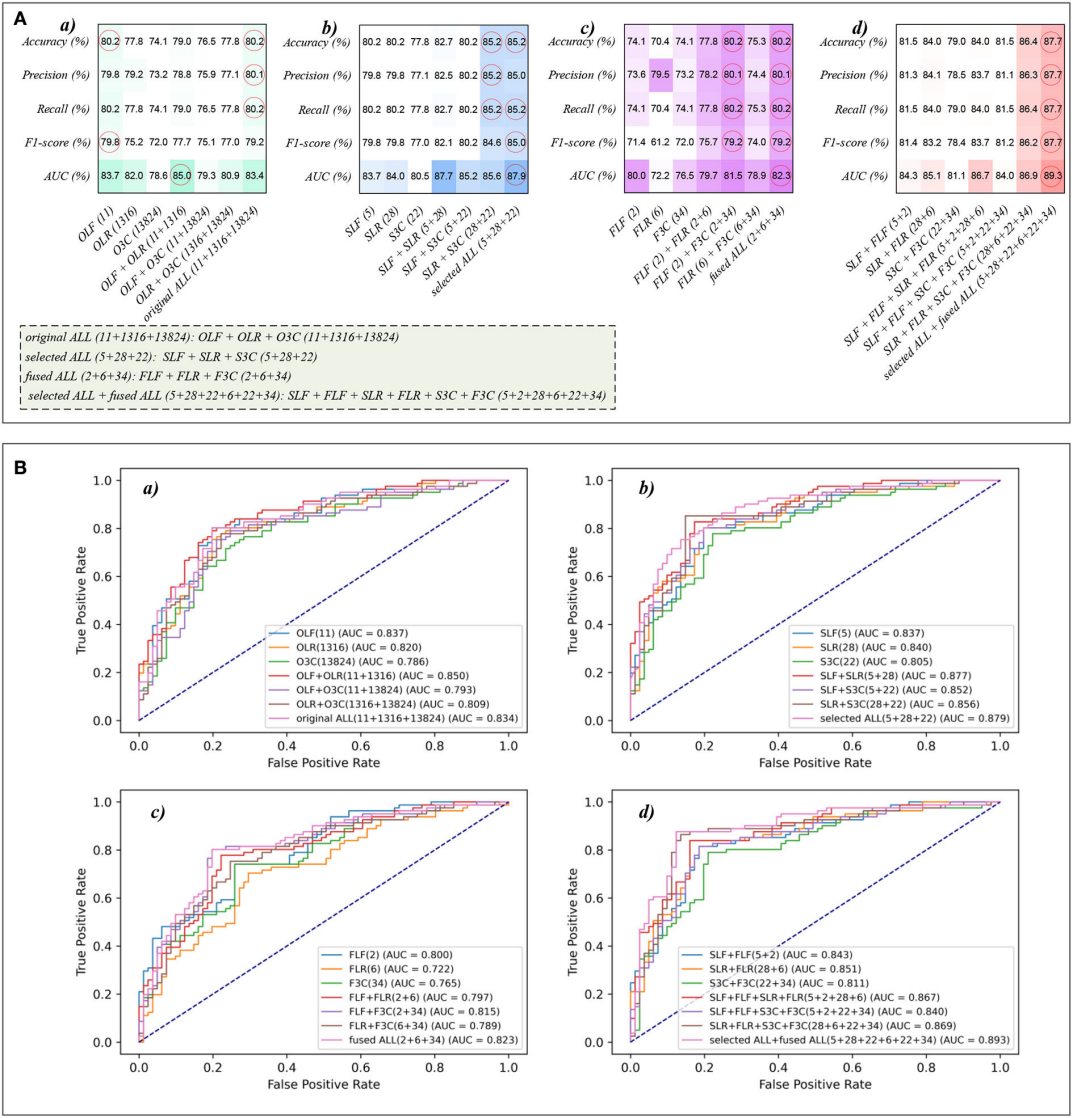
The evaluation metrics pictures and ROCs of MLP classifiers with different features. **(A)** The evaluation metrics pictures of MLP classifiers in **(a)** Experiment 1, **(b)** Experiment 2, **(c)** Experiment 3, and **(d)** Experiment 4; **(B)** ROCs of MLP classifiers in **(a)** Experiment 1, **(b)** Experiment 2, **(c)** Experiment 3, and **(d)** Experiment 4.

Because of the MLP classifier's excellent performance in dyspnea identification, Figure 8 shows the evaluation metrics pictures and ROCs of MLP classifiers with different features in Experiments 1–4 (Figure 2). Figure 8A(a) shows that although OLF (11), OLR (1,316), and O3C (13,824) are directly combined, the classification performance of the MLP classifier is basically not improved. However, Figure 8A(b) shows that the classification performance of selected combination features based on the MLP classifier has improved. In addition, Figure 8A(c) shows that compared to the single-fused features, the classification performance of the fused combination features based on the MLP classifier has improved. However, compared with the classification performance of the original features or their combination features, the classification performance of the fused combination features based on the MLP classifier is also not improved. Finally, Figure 8A(d) shows that our proposed strategy by combining the local and global features of OLF, OLR, and O3C based on the MLP classifier performs the best classification performance, achieving 87.7% of accuracy, 87.7% of precision, 87.7% of recall, 87.7% of F1-scorel, and 89.3% of AUC.

## 4. Discussion

This paper proposes a multi-modal data combination strategy by concatenating selected and fused PFT parameters, lung radiomics features, and 3D CNN features for dyspnea identification based on the MLP classifier. This section discusses three aspects: the single original modal data, Lasso and PCA, the proposed multi-modal data combination strategy, and the MLP classifier for dyspnea identification. Last, we also point out the limitations in this study and the future direction.

### 4.1. The single original modal data for dyspnea identification

The single original modal data makes it difficult to achieve satisfactory performance of dyspnea identification in COPD for clinical application. Compared with OLR (1,316) or O3C (13,824) extracted from chest HRCT images, PFT parameters OLF (11) performs best for dyspnea identification in the mean evaluation metrics. The reason for PFT parameters achieving the best identification performance also can be explained. Compared with chest HRCT images, the PFT parameters can directly reflect the respiratory status of the lungs. Therefore, the PFT parameters perform better in dyspnea identification than OLR (1,316) and O3C (13,824). Specifically, as pulmonary function index in PFT, FEV_1_ and FEV1/FVC are the criteria for determining COPD classification (1). Therefore, they may be major factors in dyspnea in COPD. Unfortunately, because of the heterogeneity of COPD patients, some patients are without dyspnea even if they are in a higher COPD stage, such as GOLDIII&IV (Figure 1B). In addition, the alveolar wall structure is damaged in severe COPD patients, leading to alveolar fusion, which further reduces the area of the pulmonary vascular bed so that the gas exchange area is reduced. The proportion of ventilation/blood flow is an imbalance, which may lead to the decline of diffusion function. The mechanisms of exertional dyspnea in patients with mild COPD and low resting DL_CO_ have been revealed (49). The TLC, FVC, and RV increase, vital capacity decreases, and the flow rate in the respiratory process decrease in COPD patients, which may result in dyspnea (50).

### 4.2. The Lasso and PCA algorithm for dyspnea identification

The Lasso algorithm is respectively performed to select the SLF (5), SLR (28), and S3C (22) from OLF (11), OLR (1,316), and O3C (13,824). Meanwhile, the PCA algorithm is respectively performed to select the FLF (2), FLR (6), and F3C (34) from OLF (11), OLR (1,316), and O3C (13,824). All ML Models based on the SLF (5), SLR (28), and S3C (22) respectively perform better than the OLF (11), OLR (1,316), and O3C (13,824) in the mean evaluation metrics. However, The FLF (2) and FLR (6) respectively perform poorer than the OLF (11) and OLR (1,316) in the mean evaluation metrics. The mean evaluation metrics of F3C (34) and O3C (13,824) basically remain unchanged. Lasso algorithm selects the identification features by establishing the relationship between the independent and dependent variables (OLF (11)/ OLR (1,316)/ O3C (13,824) and dyspnea identification), reducing the complexity of the ML classifiers and avoiding overfitting (3). While reducing the complexity of the ML classifiers, the ML classifiers respectively focus on the SLF (5), SLR (28), and S3C (22), improving the classifiers' performance for dyspnea identification. PCA algorithm fuses the identification features by reducing the dimension of the high-dimensional original features within a certain range of information loss (36). The PCA algorithm performs better at O3C (13,824) than the OLF (11) and OLR (1,316). Specifically, the OLF (11) and OLR (1,316) are not high-dimensional features. Therefore, certain identification information is lost when the original features of OLF and OLR's dimensionality reduction are performed. The mean evaluation metrics of F3C (34) and O3C (13,824) remain unchanged, confirming the discussion about the PCA algorithm above.

### 4.3. The proposed multi-modal data combination strategy for dyspnea identification

The main problem of the multi-modal data combination is that a smaller number of features OLF (11) are overwhelmed by a larger number of features OLR (1,316) and O3C (13,824). Therefore, the mean evaluation metrics of the ML Models based on original combination features have not been improved. Inspired by YOLOv3-SPP (51) (a CNN for the target detection), a multi-modal data combination strategy is proposed by combining the local and global features for dyspnea identification in COPD. The Lasso algorithm, with excellent performance for COPD dyspnea identification and its discussion above, is used to obtain the local features. Meanwhile, the global features are obtained by the PCA algorithm. The PCA algorithm fails to improve the identification performance, but the mean evaluation metrics of the local and global features have been improved. One important reason is that we select and fuse the original features separately and combine them for dyspnea identification. The local features are relevant for identifying dyspnea, but in any case, other possible features have been ignored by the Lasso algorithm. However, global features are obtained by the PCA algorithm, fusing all original features, which makes up for the defects of local features. Further, the advantages of PFT and CT are fully exploited. Except for PFT parameters and lung radiomics features, deep 3D CNN features are extracted from chest HRCT images. The local and global features of the PFT parameters, lung radiomics features, and 3D CNN features are re-integrated, finally obtaining a good dyspnea identification effect.

### 4.4. The MLP classifier for dyspnea identification

Eight classical ML classifiers are respectively used for dyspnea identification in COPD. The MLP classifier performs better than the other classifiers in this paper, implying that there may be a non-linear relationship between identification features and dyspnea. In addition, due to the multi-modal data combination, there are essential differences between the multi-modal features. In particular, the OLF (11) is obtained by PET, and OLR (1,316) and O3C (13,824) are extracted from chest HRCT images imaged by CT. The MLP classifier with strong adaptive and self-learning ability can handle the multi-modal data combination well. Meanwhile, 13,824 3D CNN features are the non-linear classification features. The MLP classifier is good at handling complex non-linear features by itself, which fits the essence of the MLP classifier and is interpretable (3, 37).

### 4.5. The limitations in this study and future direction

This study also has some limitations, and we point out the future direction. First, the number of our study cohort limits the multi-classification of dyspnea in COPD, which may be more meaningful in clinical COPD management. Second, dyspnea in COPD is identified only by engineering means. However, professional clinicians should further analyze the deeper relationship between dyspnea and identification features from a pathophysiological point of view. Third, the existing classic ML classifiers are not improved. Last, the measurement of PFT parameters is very complex and limited by the cooperation of the examiner (52). In our future work, the improved graph neural network, an auto-metric Graph Neural Network based on a meta-learning strategy (12, 53), will be further attempted and modified for dyspnea identification. Meanwhile, this paper only uses chest HRCT images and PFT parameters for dyspnea identification. Other clinical information should be collected to further improve dyspnea's classification performance, such as the heterogeneity parameter ventilation/perfusion $V^{\prime }$/Q' is a major contributor to dyspnea in COPD patients (54, 55). Besides, the mMRC score of 1 is a rather low dyspnea level and can even be physiological breathlessness in older subjects. However, identifying severe and extremely severe dyspnea in COPD may be more valuable for clinical application. Therefore, in subsequent studies, we will further expand our research to reveal the rule of dyspnea in COPD with aging using a survival analysis model.

## 5. Conclusions

This paper proposes a multi-modal data combination strategy by combining the local and global features for dyspnea identification in COPD based on the MLP classifier. Specifically, the Lasso algorithm is separately performed to select the local features from original multi-modal data (11 original PFT parameters, 1,316 original lung radiomics features, and 13,824 original 3D CNN features). Meanwhile, the PCA algorithm is separately performed to fuse original multi-modal data, generating the global features. All the local and global features of original multi-modal data are combined for dyspnea identification in COPD based on the MLP classifier, achieving the best classification performance at 87.7% of accuracy, 87.7% of precision, 87.7% of recall, 87.7% of F1-score, and 89.3% of AUC, respectively. Compared with single-modal data, our proposed multi-modal data combination strategy effectively improves the classification performance for dyspnea identification in COPD, providing an objective and effective tool for COPD pre-clinical health management.

## Data availability statement

The original contributions presented in the study are included in the article/Supplementary material, further inquiries can be directed to the corresponding authors.

## Ethics statement

The studies involving human participants were reviewed and approved by National Clinical Research Center of China's respiratory diseases. The patients/participants provided their written informed consent to participate in this study.

## Author contributions

Conceptualization and supervision: YK and RC. Methodology: YY, ZC, WL, and YG. Software: YY, ZC, NZ, SW, and WD. Validation: WL, YY, ZC, XL, and HC. Formal analysis: YY, ZC, YL, WL, and YG. Investigation: HC and XL. Resources: HC, RC, and XL. Data curation: RC. Writing—original draft preparation: YY and ZC. Writing—review and editing: WL, YL, and YK. Visualization: ZC, YY, YL, NZ, SW, ZC, and WD. Project administration: YK and HC. Funding acquisition: YK, WL, and HC. All authors have read and agreed to the published version of the manuscript.
