# Supplementary material for: Multi-modal data combination strategy based on chest HRCT images and PFT parameters for intelligent dyspnea identification in COPD
Source: Front Med (Lausanne). 2022 Dec 21;9:980950. doi: 10.3389/fmed.2022.980950 (PMC9811121; doi:10.3389/fmed.2022.980950)
Supplement: Supplementary file 1 [file Data_Sheet_1.ZIP › Supplementary Materials V2_20221129/Table S8. The definitions and parameters of the eight classifiers.docx]

**Table S2.** The definitions and parameters of different classifiers.

| **Classifier** | **Model definition in Python 3.6** |
| --- | --- |
| SVM | SVM sklearn.svm.SVC(kernel=‘rbf’,probability=True) |
| MLP | sklearn.neural_network. MLPClassifier (hidden_layer_sizes=(400, 100), alpha=0.01, max_iter=10000) |
| RF | sklearn.ensemble.RandomForestClassifier(n_estimators=200) |
| KNN | KNN sklearn.neighbors. sklearn.neighbors() |
| LR | sklearn.linear_model.logisticRegressionCV(max_iter=100000, solver="liblinear") |
| GB | sklearn.ensemble.GradientBoostingClassifier() |
| LDA  LR | sklearn.discriminant_analysis.()  LR sklearn.linear_model.logisticRegressionCV(max_iter = 100,000, solver = “liblinear”) |
